# Investigation of MicroRNA-21 Expression Levels in Serum and Stool as a Potential Non-Invasive Biomarker for Diagnosis of Colorectal Cancer

**DOI:** 10.18869/acadpub.ibj.21.2.106

**Published:** 2017-03

**Authors:** Saiyad Bastaminejad, Morovat Taherikalani, Reza Ghanbari, Akbar Akbari, Nooshin Shabab, Massoud Saidijam

**Affiliations:** 1 Department of Molecular Medicine and Genetics, Research Center for Molecular Medicine, School of Medicine, Hamadan University of Medical Sciences, Hamadan, Iran; 2 Razi Herbal Medicines Research Center, Department of Microbiology, School of Medicine Lorestan University of Medical Sciences, Khorramabad, Iran; 3 Digestive Oncology Research Center, Digestive Disease Research Institute, Tehran University of Medical Science, Tehran, Iran; 4 Abadan School of Medical Sciences, Abadan, Iran

**Keywords:** Serum, Stool, miR-21, Biomarker, Colorectal Cancer (CRC)

## Abstract

**Background:**

Most cancer studies focus on exploring non-invasive biomarkers for cancer detection. In the present study, we sought to investigate the expression level of microRNA-21 (miR-21), as a potential diagnostic marker, in serum and stool samples from 40 patients with colorectal cancer (CRC) and 40 healthy controls.

**Methods:**

Quantitative real-time RT-PCR was applied to determine the relative expression level of miR-21 in serum and stool. At the same time, the sensitivity and specificity of this marker was evaluated by receiver operating characteristic (ROC) curve analysis.

**Results:**

miR-21 expression levels of serum and stool were up-regulated 12.1 (*P*<0.05, 95% CI: 5.774-34.045) and 10.0 (*P*<0.05, 95% CI: 0.351-16.260) times in CRC patients, respectively, when compared to the control group. The sensitivity and specificity of miR-21 was found to be 86.05% and 72.97%, respectively (an area under the ROC curve [AUC] of 0.783). The stool miR-21 level in CRC patients was much higher than that in the healthy controls, showing a sensitivity of 86.05% and a specificity of 81.08% (AUC: 0.829). The expression level of miR-21 in stool was able to significantly distinguish CRC tumor, node, metastasis stages III-IV from stages I-II, with a sensitivity and specificity of 88.1% and 81.6%, respectively (AUC: 0.872).

**Conclusion:**

The results of this study indicated that miR-21 expression levels in serum and stool can be considered as a potential diagnostic biomarker for the diagnosis of CRC patients. However, more studies are required to confirm the validity of miR-21 as a valuable non-invasive diagnostic tool for CRC.

## INTRODUCTION

Nowadays, non-invasive biomarkers are being widely explored as a reliable tool for cancer diagnosis. Colorectal cancer (CRC) is the second leading cause of cancer death in the world[1,2]. Typically, fecal occult blood test (FOBT) and serum carcinoembryonic antigen (CEA) levels are measured to screen CRC patients in primary stages. These methods are not specific for CRC diagnosis and should be supplemented by pathology reports. The tumor, node, metastasis (TNM) staging system is based on the pathologic findings and generally applied to describe the stage of cancer. In addition, the disadvantages of invasive and difficult sample collection have limited the application of pathological methods in the diagnosis of CRC. More recently, increasing attention has been paid to serum and fecal markers to detect CRC and other malignancies[3,4]. Currently, molecular methods, as transcriptional profiling of gene expression, have been widely used to determine prognosis, evaluate therapeutic response and assess CRC tumor grading[5].

CRC is a multi-stage process specified by genetic and epigenetic variations that affect the main cellular pathways involved in growth and development[6]. Therefore, a better understanding of molecular mechanisms of CRC development may offer new avenues to exploit potential prognostic biomarkers and therapeutic targets for CRC[7]. The early detection of cancer significantly improves the overall survival rate of patients, thus emphasizing the need for finding new biomarkers for early-stage detection of CRC[8,9]. MicroRNAs (miRNAs), as a potential candidate for early diagnosis of cancer, can be detected in tissue, feces and serum[10-12]. MiRNAs are small single-stranded RNAs of 18-24 nucleotides in length that play an important role in gene regulation. These molecules exert their effects by binding to the 3’-untranslated region of their target mRNAs to negatively regulate their expression[13,14]. MiRNAs, as an oncogene or tumor suppressor, can be down-regulated or up-regulated in tumor tissues as compared with normal tissues[15-16].

MiRNA-21 is an oncogenic miRNA that regulate the expression of certain target genes such as programmed cell death 4, phosphatase and tensin homologue and tropomyosin 1[17]. Recent studies have shown that this miRNA is up-regulated in CRC and many other malignancies[18-21]. Nevertheless, the majority of these studies have been conducted in tissue or serum samples, but not in stool samples. During CRC development, a large number of tumor cells are exfoliated into feces, serving as a source of released miRNAs in stool[22,23]. In view of the above explanations, the aim of this research was to evaluate the miR-21 expression level using real-time quantitative RT-PCR (qRT-PCR), in order to elucidate the clinical significance and potential efficiency of miR-21 as a valuable biomarker.

## MATERIALS AND METHODS

### Subjects and sample collection

Totally, 40 CRC patients and 40 healthy controls were participated in this study (Table 1). Blood and stool samples were obtained from Shariati Hospital, Tehran, Iran, in the period from 2014 to 2015. A written informed consent was taken from all participants, and ethical approval was obtained from the Ethical Committee of Hamadan University of Medical Sciences (Hamadan, Iran). Detailed exclusion and inclusion criteria applied for the patients and controls are indicated in Table 2.

**Table 1:** Healthy controls’ and Patients’ clinical characteristics

Characteristics	Healthy controls	Patients
Age (mean:54, SD:9.4)		
≤50	16	18
>50	24	22
Gender		
Male	21	21
Female	19	19
TNM staging		
I	11	
II	16	
III	6	
IV	7	
Tumor type		
Colon	22	
Rectum	18	
Tumor size (cm)		
≤5	24	
>5	16	
Tumor location		
Right side	19	
Left side	21	

The data were collected from patients’ files in Shariaeti Hospital (Tehran, Iran). TNM, tumor-node-metastasis

**Table 2:** Inclusion and exclusion criteria to determine the study eligibility

Criteria	Patients	Healthy controls
Inclusion	-Malignant or precancerous confirmed by colonoscopy and histopathologic examination	-No history of colorectal cancer or adenoma -No positive fecal occult blood test or FIT during the previous six months -No previous colorectal resection for any reason other than sigmoid diverticular disease -No IBD including CUC and Crohn’s disease
Exclusion	-No hospital records	-A history of colorectal cancer or adenoma -Positive fecal occult blood test or FIT during the previous six months -Previous colorectal resection for any reason other than sigmoid diverticular disease -IBD including CUC and Crohn’s disease

CUC, chronic ulcerative olitis; IBD, inflammatory bowel disease; FIT, fecal immunochemical test

### Serum and stool collection and RNA extraction

Prior to surgical tumor resection and seven days after colonoscopy, whole-blood samples were collected from each participant into 5-ml RNase-free tubes. The serum was then separated and stored at -80°C until use. Stool samples were collected simultaneously from the both groups as the whole blood samples, immediately flash frozen in liquid nitrogen, and stored at -80°C. On the sampling day, the following basic demographic and clinical information was collected from all participants: cancer stage at diagnosis, the histological type and the grade of the tumor. The staging of tumors was determined according to the American Joint Committee on Cancer TNM staging system[24,25]. It should be noted that the quality of total RNA extracted from prototypes is extremely important[26]. In this light, the miRNeasy serum/plasma (Cat number:Q217184; Qiagen, Germany) and miRNeasy mini kits (Cat number: Q217004; Qiagen, Germany) were used to extract miRNAs from serum and stool samples, respectively. The total amount of stool samples used for this kit was 100 mg. The miRNeasy serum/plasma kit is able to efficiently purify RNA from serum or plasma. Using miRNeasy Kits, it is possible to provide highly pure RNA suitable for downstream applications.

More importantly, miRNeasy procedures minimize the possibility of contamination with salts or phenol, which could interfere with later analyses. The total amount of serum samples used for this kit was 200 µl. Total miRNAs were extracted according to the manufacturer’s instructions. The concentration and purity of the extracted miRNAs were assayed using the Eppendorf BioPhotometer (Eppendorf AG, Hamburg, Germany).

### cDNA synthesis and real-time quatitative RT-PCR

Locked nucleic acid (LNA) kits and primers (Exiqon, Germany) were used for cDNA synthesis and real-time qRT-PCR detection. LNA kits have been proved to be the most suitable form of miRNA amplification[27,28]. cDNA was synthesized using the miRCURY LNA Universal cDNA synthesis kit II, 8-64 rxns (cat number: 203301; Exiqon, Vedbaek, Denmark). qRT-PCR was performed by ExiLENT Syber® Green Master Mix Kit (cat number:203403; Exiqon, Germany). MiR-16, RNU6B (U6 small nuclear RNA), and miR-21-specific LNA™ PCR primer sets (Exiqon, Germany) were applied according to the manufacturer’s instructions. The qRT-PCR reactions were carried out using a CFX96 Real-Time PCR system (Bio-Rad, Milan, Italy) under the following conditions: an initial denaturation at 95°C for 10 min, followed by 48 cycles of denaturation at 95°C for 10 sec, and annealing and extension at 60°C for 1 min at a ramp rate of 1.6°C/s. The specificity and identity of the PCR products were verified by melting curve analysis after the last amplification cycle. To ensure the reproducibility and fidelity of the results, all samples were run in duplicate. As a pilot study, the expression of RNU6B and miR-16 were examined as reference genes before normalization. Until now, there has been no reliable internal control for stool-based miRNAs. Due to a relatively stable expression level, miR-16 was used as a reference housekeeping gene for data normalization in serum and stool examinations. The 2^-ΔΔCT^ method [ΔΔCT=ΔCT (a miRNA of interest)-ΔCT (miRNA-16 as a normalizer accounting for sample-to-sample variation)] was used to analyze the relative expression of this miRNA[28,29]. In addition, UniSp6 Spike-in was used in this study as a positive control, where the UniSp6 Spike-in template and UniSp6 Spike-in control primer set were applied for cDNA synthesis and real-time PCR detection, respectively.

### Statistical analysis

Tables 1 and 2 show the patients and healthy control subjects adjusted for potential confounding factors such as demographic variables and tumor characteristics. Based on relative expression of miR-21 as a target gene versus miR-16 as a reference gene, the up-regulated expression of miR-21 was measured using the ΔΔCT method[29] and Relative Expression Software Tool 2009 (Qiagen, Hilden, Germany). The Analyses of variance (ANOVA), Kruskal-Wallis, and Mann-Whitney statistic tests were used to evaluate the differences of miRNA expression levels in serum or stool samples. As a diagnostic test, Receiver Operating Characteristic (ROC) curve analysis was applied to determine the sensitivity and the specificity of miR-21 expression in serum and stool of CRC patients. In addition, internal validation was performed using the BCa bootstrap method to accurately estimate the ROC curves and optimal cut-off values. The method was used to discriminate CRC patients from healthy control subjects, as well as patients with TNM stages III-IV from stages I-II. Data analysis was performed by using MedCalc®version 13.1.2.0 (Acacialaan 22, 8400 Ostend, Belgium).

## RESULTS

### miR-21 expression levels in serum and stool

**Fig. 1:**
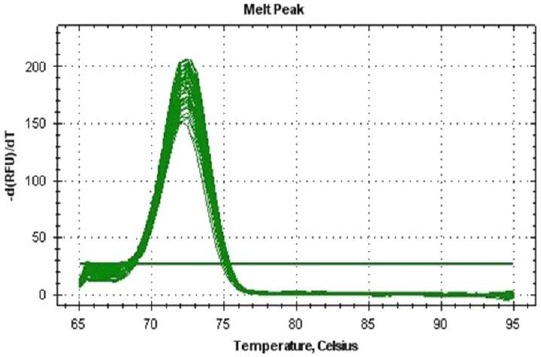
qPCR Melting Curve for serum miR-21 levels in CRC patients. The miR-21 amplicon shows a single peak, representing a pure, single amplicon.

After checking the specific amplification (Fig.1), the miR-21 expression level was determined in both serum and stool samples from CRC patients and healthy control subjects. In comparison to the control group, the miR-21 expression levels in serum and stool in CRC patients were up-regulated 12.1 (*P*<0.05, 95% CI: 5.774-34.045) and 10.0 times (*P*<0.05, 95% CI: 0.351-16.260), respectively. A significant association was found between the miR-21 expression level in stool and serum of CRC patients using the Mann-Whitney statistical test (*P*<0.05).

### miR-21 expression levels in serum and stool based on patients’ clinical characteristics

**Fig. 2:**
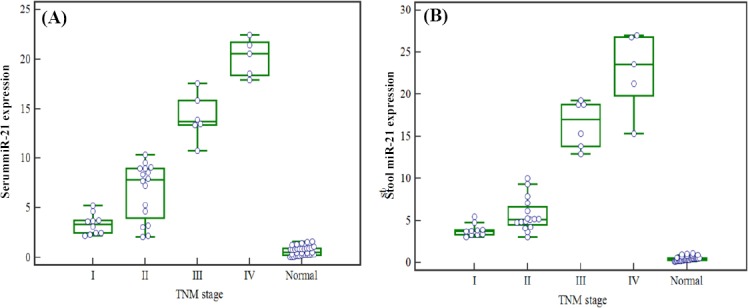
miR-21 expression level in serum and stool based on patients’ clinical TNM stages. Results of kruskal-wallis test show that A) Serum miR-21 levels represent no obvious increase in patients with stage IV, ascompared to those with stage III (*P*>0.05), while a significant increase was found between patients with stages I and II (*P*<0.05); B)The expression level of miR-21 in stool can be considered as a promising marker to distinguish TNM stages III and IV from stages I or II (*P*<0.05), while unable to discriminate stage III from stage IV (*P*>0.05).

TNM staging criteria revealed that there was no significant increase in the expression of miR-21 in serum and stool from patients with stage IV CRC, when compared to those with stage III CRC (*P*>0.05). However, the expression level of miR-21 in patients with stage III CRC was significantly higher than those with stages I and II (*P*<0.05) (Fig. 2). No significant difference was observed in the expression level of miR-21 in serum and stool of CRC patients according to age and gender as well as tumor type, size, and location (*P*>0.05) (Table 3).

**Table 3:** miR-21 expression levels in serum and stool based on patients’ clinical characteristics

Characteristics	miR-21 level in serum		miR-21 level in stool	
	
	mean ± SD	*P* value	mean ± SD	*P* value
Age						
≤50	11.20±0.58	0.91	10.3±0.35	0.12
>50	11.32±0.41		9.24±0.43	
Gender						
Male	10.94±0.61	0.90	8.76±0.71	0.91
Female	11.40±0.23		9.94±0.15	
Tumor type						
Colon	11.8±0.17	0.86	10.2±0.23	0.74
Rectum	11.1±0.46		9.83±0.72	
Tumor size (cm)						
≤5	12.0±0.13	0.77	11.2±0.48	0.06
>5	11.6±0.55		9.61±0.44	
Tumor location						
Right side	10.58±0.38	0.98	11.20±0.73	0.71
Left side	11.74±0.17		10.64±0.37	

There is no significant association between stool and serum miR-21 expression levels and clinical characteristics (age, gender, as well as tumor type, size, and location) in CRC patients (*P*>0.05).

### miR-21 as a non-invasive biomarker for CRC diagnosis

**Fig. 3:**
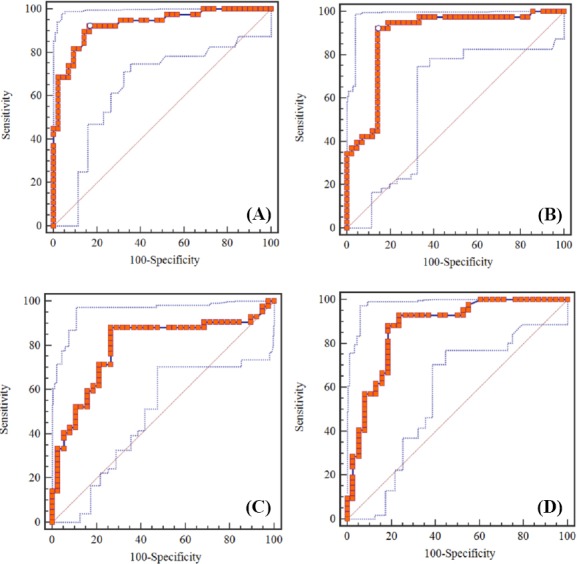
Receiver operating characteristic (ROC) curve analysis to evaluate serum and stool miR-21 expression levels for the detection of CRC patients. A) Serum miR-21 level for the detection of CRC patients; the area under the ROC curve (AUC): 0.783, Youden index: 0.590, sensitivity: 86.05%, and specificity: 72.97%; B) Stool miR-21 level for the detection of CRC patients, AUC: 0.829, Youden index: 0.671, sensitivity: 86.05%, and specificity: 81.08%; C)Serum miR-21 expression level for the detection of tumor, node, metastasis (TNM) stages in CRC patients, AUC: 0.794, Youden index: 0.617, sensitivity: 88.10%, and specificity: %73.68; D) Stool miR-21 expression level for the detection of TNM stages in CRC patients, AUC: 0.872, Youden index: 0.696, sensitivity: 88.10%, and specificity: 81.58%.

As shown in Figure 3, ROC curves were developed to evaluate the diagnostic potential of miR-21 as a non-invasive biomarker candidate. ROC curve analysis indicated that the serum miR-21 expression level could be considered as a promising marker for the diagnosis of CRC patients with a sensitivity and specificity of 86.05% and 72.97%, respectively (an area under the ROC curve, AUC: 0.783). In addition, the sensitivity and specificity of miR-21 in stool samples were 86.05% and 81.08% (AUC: 0.829), demonstrating its ability to discriminate CRC patients from healthy controls. No significant differences were observed between serum and stool levels (*P*<0.05). The ROC analyses also showed that serum miR-21 expression levels were able to reliably distinguish TNM stages III and IV from stages I and II, with a sensitivity of 88.10% and a specificity of 73.68% (AUC: 0.794). Furthermore, differences in the stool miR-21expression level could successfully discriminate CRC TNM stages III-IV from stages I-II, with a sensitivity and specificity of 88.1% and 81.6%, respectively (AUC: 0.872). Pathologic findings are generally considered as a gold standard for the diagnosis of CRC and the evaluation of TNM stages. The optimal threshold cut-off values were determined at the point on the ROC curve, at which Youden’s index (=sensitivity+[100%−specificity]) was maximum. The optimal cut-off values were used to calculate the sensitivity and specificity as well as positive and negative predictive values. The cut-off value estimated for serum and stool miR-21 levels was 1.1 and 3.05, respectively.

## DISCUSSION

Molecular techniques, especially gene expression profiling, are commonly used to improve CRC classification, elucidate patients’ prognosis and predict response to therapy[27,28]. Early detection of tumor improves the overall survival rate of CRC patients, which highlights an urgent need to find specific, sensitive and non-aggressive molecular biomarkers suitable for the early diagnosis of CRC[30,31]. In recent years, there has been an increased interest in finding prognostic biomarkers for CRC to evaluate the expression profiles of single or multiple miRNA in tumor tissues[31,32].

To date, several interesting miRNAs have been candidated as CRC molecular biomarkers; however, it is difficult to draw a definite conclusion[33-35]. The presence of miRNAs in different tissues as well as in feces, urine, and serum makes them excellent targets for development of potential molecular biomarkers[36-38]. In line with previous investigations[39-41], the findings of the present study demonstrated that miR-21 is up-regulated in serum and stool samples from CRC patients. No statistically significant difference was found between miR-21 expression levels in serum and stool samples. Based on the results of this study, it can clearly be found that the miR-21 expression level in serum, stool, and tumor tissues of CRC patients was correlated with its expression in tumor cells. This possibility is supported by the fact that previous studies have shown that miR-21 expression levels are significantly increased in tumor tissues[5,41]. Interestingly, no significant relationship was found between miR-21 expression levels and patients’ clinical and demographic characteristics, including age, gender, and tumor location.

Previous studies have indicated that the miR-21 expression level is increased in different TNM stages of CRC, from the early to later stages[31,33]. In the present study, no obvious differences were detected in miR-21 expression levels between stages III and IV, while a significant increase was found between stages I and II. This difference could be attributed to the number of cases studied in each stage. The sensitivity and specificity of miR-21 expression levels, as a CRC molecular biomarker, were assessed in serum and stool of CRC patients using ROC curve analysis. As mentioned above, miR-21 expression levels in both serum and stool showed a reasonable sensitivity and specificity to diagnose CRC patients and determine TNM stages. In a study carried out by Toiyama *et al*.[31]3, it turned out that the sensitivity and specificity of serum miR-21 expression levels were 82.8% and 90.6% for CRC detection, respectively. It is important to note that if the cancer is detected at early stages (I and II), the survival rate increases. Considering this fact, using ROC analysis, the sensitivity and specificity of miR-21 expression was assessed in serum and stool of CRC patients to distinguish stages I and II from stages III and IV. Using Medcalc statistical software, comparison between ROC curves showed no statistically significant difference between the sensitivity and specificity of this miRNA in serum and stool. The results of this study suggested that miR-21 expression level in serum and stool could be considered as a valuable marker for CRC diagnosis. However, more studies with larger sample sizes are required to confidently confirm our findings. The design of an appropriate internal control is the most important achievement to normalize real-time PCR data[29]. In most previous studies, miR-16 and RNU6B were used as an internal control[42,30-34]. Since there is no exact recommended miRNA as a reference gene[31], we attempted to test both miRNAs for defining reference genes. Surprisingly, it was found that miR-16, as an internal control, is more suitable than RNU6B due to its greater stability.

In summary, serum and stool miR-21 expression levels seem to be a potential diagnostic molecular biomarker for CRC patients. However, well-designed studies with larger sample sizes are recommended to further investigate the role of miR-21 in CRC diagnosis.
